# Scraps

**Published:** 1899-09

**Authors:** 


### SCRAPS

PICKED UP BY THE ASSISTANT-EDITOR.

---

**Clinical Records** (28).—This conversation is said to have taken place in the out-patient department of an East-end hospital:—House-Surgeon: "Come, now, tell the officer who did it. Was it your husband?" Patient: "No, he wouldn't a done it—he 's more like a friend than a husband!"

**Modern Therapeutics.**—*The Post-Graduate* says that those who fear that since the times of Addison and Macaulay, the age of fine writing is past, may comfort their anxious souls, by reading the following extract from a medical paper in a recent number of one of our great journals:

"*He who has watched for the past decade that jostling horde of barbaric drugs which, emerging from the sulphurous mists of the chemist's laboratory, have swept across the medical horizon in unbroken column, only to disappear in the silent caverns of oblivion, will hardly care to attack the statement that permanency is not the most obtrusive characteristic of prevailing methods of treatment.*"

**Provincial Medical Schools.**—Writing on the advantages of provincial medical schools, the *Medical Press and Circular* says: "Among the smaller provincial schools, Bristol, one of the oldest, has always been distinguished for the quality of its teaching. Of late years the status of the school has been materially advanced by its affiliation with the University, and the erection of handsome new buildings. Quite recently another great step has been taken to increase the facilities of students in obtaining clinical material. The Royal Infirmary, dating from ancient times, has lately joined forces with the more modern Bristol Hospital, so far as the training of students is concerned. A large surgical practice is always to be witnessed at both institutions, owing to the position of the town as a large manufacturing centre and seaport."

**Examination Papers.**—The following are taken from some reported answers:—

The stomach is the most diluted portion of the elementary canal.

Hygiene is all that you can tell about that which is asked.

The doctrine of evolution began with the beginning of life, and grew higher and higher, until it regenerated into monkey. This process was slow—so slow that neither the monkey nor the man knew anything about it.

A germ is a name applied to a particular particle, tiny subbacterial organism, which, when demonstrated, causes disease.

A germ is a tiny insect sometimes found in diseases or organs—that is why diseases are contagious. It is so small that it can be seen only with a telescope. Then it appears like the head of a pin, but it goes floating around into the atmosphere.

The germ theory of diseases is continually floating around in the air, and is very dangerous, especially when the atmosphere is unwholesome.

**The Experience of an Army Surgeon.**—*The Doctor's Factotum* says that the following verses, dedicated to the editor of the *Medical Standard*, were read by Dr. Jay at the Phi Rho Sigma Interchapter "Smoker," at the Grand Pacific Hotel banquet-hall. Dr. Jay assures the editor that the verses contain more truth than poetry:

"Some of these dysenteric germs
Into my food had stolen,
And soon were where they love to roam—
Within a human colon.
They thought my cæcum should be red,
And this they did inflame;
They discovered my appendix,
And they colonised the same.

"They travelled through the sugar
Down in the crypts of Lieberkühn
Explored the Peyer's patches,
And more ambitious soon,
They dug in the mucosa,
Where they licked the phagocytes,
As the cowboys licked the Spaniards
Upon El Caney heights.

"Then over to my sigmoid
Some millions emigrated
And everywhere my colon
They with ulcers decorated.
They dug those ulcers deeper
Than the muco-fibrous juncture,
And every time I had the cramp
I thought it was a puncture."

**Obstetrics Home and Foreign** (6).—"In the Injunctions at the Visitation of Edmunde (Bonner) Bishop of London from September 3d, 1554, to October 8th, 1555, 4to., we read: 'A mydwyfe (of the diocese and jurisdiction of London) shal not use or exercise any witchecrafte, charmes, sorcerye, invocations or praiers, other than suche as be allowable and may stand with the lawes and ordinances of the Catholike Churche.'

"In the Articles to be enquired in the Visitacyon in the fyrst yeare of Queen Elizabeth, 1559, the following occurs: 'Item, whether you knowe anye that doe use charmes, sorcery, enchauntmentes, invocations, circles, witchecraftes, southsayinge, or any lyke craftes or imaginacions invented by the Devyl, and *specially in the tyme of women's travayle*.' It appears from Strype's Annals of the Reformation, i. 537, under 1567, that then midwives took an oath, inter alia, not to 'suffer any other bodies child to be set, brought, or laid before any woman delivered of child, in the place of her natural child, so far forth as I can know and understand. Also I will not use any kind of sorcery or incantation in the time of the travail of any woman.'"—Brand's *Popular Antiquities*, ed. Bohn, 1849, vol ii., p. 69.

"The *Yi* of India, the *Dye* of Syria, the herb-knowing hag of Mexico, and the midwife of the Bible are very much the same in their habits, their qualifications, and their knowledge. It is the same habitual old woman who figures in all countries and at all times, and with whose peculiar qualifications we are quite familiar. In cases where the midwife is at a loss, the aid of the medicine man is sought. The Baschkirs rely upon their 'devil-seer,' who discovers the presence of the evil spirit and drives him away if rewarded by the present of a sum of money or a fat sheep. Among others a priest is called who hastily mumbles a few verses of the Koran, spits into the patient's face, and leaves the rest to nature."—Engelmann's *Labor Among Primitive Peoples*, 1882, p. 131.

**Medical Philology** (XXXI.).—The *Promptorium* gives "Dyndelyñ, *Tinnio*," and the *Catholicon Anglicum* "to Dindylle. *Condolere*" or "*Errobare*."

In his note, Mr. Way points out that these are somewhat different senses, the *Catholicon* equivalent signifying "suffering acutely." He adds: "Brockett gives to dinnel, or dindle, to be affected with a pricking pain, such as arises from a blow, or is felt by exposure to the fire after frost. In the Craven dialect to dinnle has a similar signification. Langham, in the Garden of Health, 1579, recommends the juice of feverfew as a remedy for the 'eares ache, and dindling.' Dutch, tintelen, to tingle."

Mr. Herrtage in his *Catholicon* note says: "In Jamieson we find 'To dinle, dynle. (1) To tremble. (2) To make a great noise. (3) To thrill; to tingle.' 'Dinle, *s.* (1) Vibration. (2) A slight and temporary sensation of pain, similar to that caused by a stroke on the elbow.' Cotgrave gives '*Tintillant.* Tinging; ringing; tingling, *Tintoner.* To ting or towle often; to glow, tingle, dingle.' 'Hir unfortunat husband had no sooner notice given him upon his returne of these sorrowfull newes than his fingers began to nibble . . . his ears to *dindle*, his head to dozell, insomuch as his heart being scared with gelousie . . . he became as mad as a March hare.'—Stanihurst, Descrip. of Ireland in Holinshed's Chronicles (1576), vol. vi. p. 32, § 2.

'The birnand towris doun rollis with ane rusche,
Quhil all the heuynnys *dynlit* with the dusche.'
Gawin Douglas, *Eneados*, Bk. ix. p. 296, l. 35."

The *New English Dictionary* says that the derivation of the word is obscure, and that it is probably onomatopoeic. It will be seen from the references to Jamieson that it is well known as a Scottish word. Some of the quotations given above show that in some instances it had a special medical significance.

In June, 1894, I quoted from a note in Mr. Way's edition of the *Promptorium* a prescription containing feverfew, which Langham recommends as a remedy for the dindling. Feverfew has been for ages the popular name of *Pyrethrum Parthenium*, which was also called *Matricaria* because, as Turner says, "The new writers hold . . . that feverfew is better for weomen." (*Herbal*, ii. 796, quoted by the *New English Dictionary*.)

